# Hysterosalpingography findings of female partners of infertile couple attending fertility clinic at Lagos University Teaching Hospital

**DOI:** 10.11604/pamj.2021.40.223.29890

**Published:** 2021-12-14

**Authors:** Christian Chigozie Makwe, Aloy Okechukwu Ugwu, Oyebola Halimah Sunmonu, Salimat Abisoye Yusuf-Awesu, Nneoma Kwemtochukwu Ani-Ugwu, Olayemi Emmanuel Olumakinwa

**Affiliations:** 1 Department of Obstetrics and Gynecology, College of Medicine, University of Lagos, Lagos, Nigeria,; 2 Department of Obstetrics and Gynecology, Lagos University Teaching Hospital, Lagos, Nigeria,; 3 Department of Medicine, Lagos University Teaching Hospital, Lagos, Nigeria

**Keywords:** Hysterosalpingography, tubal patency, female infertility, Lagos, Nigeria

## Abstract

**Introduction:**

Hysterosalpingography (HSG) is an outpatient fluoroscopic method for the evaluation of the uterine cavity, fallopian tubes, and the surrounding peritoneal cavity. Female fertility depends greatly on normal female reproductive organs; hence tubal abnormalities may contribute significantly to female infertility. HSG is an invaluable screening tool in the evaluation of women with suspected tubal factor infertility. This study aims to review the HSG findings of women who sought fertility treatment at the Lagos University Teaching Hospital, Lagos (LUTH).

**Methods:**

this was a retrospective study of the pattern of HSG findings among female partners of infertile couples seeking fertility treatment at the LUTH, over a 2-year period, from January 2018 to December 2019.

**Results:**

a total of 266 medical records and HSG results were reviewed and included in the data analysis. The mean age (± standard deviation) was 38.4 (± 0.3) years with a range of 24 to 50 years. Most (80.5%) of the participants have secondary infertility and majority (65.4%) were nulliparous. Tubal pathology was the commonest abnormality detected on HSG in 54.9% of women. About one-third (30.8%) of women had bilateral tubal occlusion on HSG. With regards to the right fallopian tube, 43.2% of the participants had tubal occlusion, which differs from 41.7% on the left fallopian tube. Similarly, 10.2% of the women had hydrosalpinx on the left tube when compared with 9% on the right tube. Age (OR 1.055; 95% CI: 1.006, 1.106, p-value 0.028), and previous salpingectomy [OR 6.151; 95% CI: 1.335, 28.349] and myomectomy [OR 4.6; 95% CI: 1.814, 11.67] were identified as risk factors for tubal pathologies on HSG.

**Conclusion:**

tubal abnormalities are common findings on HSG and the identifiable risk factors for tubal pathologies include age, salpingectomy, and myomectomy. HSG remains a vital screening tool in the evaluation of tubal-factor infertility in Nigeria.

## Introduction

Infertility is defined as the failure to achieve pregnancy after twelve months of regular unprotected intercourse [1]. It is an issue of great concern for infertile couples, especially in Africa where a high premium is placed on childbirth and large family size. Worldwide, millions of couples are afflicted with inability to conceive or sustain pregnancy. This poses several burdens (such as economic, psychological, and emotional) to the affected family. Female factor infertility can be due to structural or functional abnormalities involving one or more of the following parts of their reproductive system: the cervix, uterus, fallopian tube, and ovary and the disorders of the endocrine syste [1-3]. Tubal damage accounts for approximately a third of female factor infertility and it is the most frequent cause of female infertility in Nigeria [3]. The prevalence of female factor of about 30 - 40% has been reported [3,4]. The prevalence of the different causes of infertility varies with the sophistication of the investigative tools available in a region, the methods/techniques used in the study, and the population evaluated. The primary goal of evaluating these infertile couples is to ascertain the likely cause of the infertility, decide on the most suitable treatment modality considering success rate, availability and affordability of the treatment protocol, and also to counsel these couples on the possible alternative treatment, if available. Hysterosalpingography (HSG) is fluoroscopic evaluation of the endocervical canal, uterine cavity, fallopian tubes and adjacent pelvic peritoneum [4,5]. The procedure can be performed using either oil or water soluble contrast media. HSG is considered an invaluable simple diagnostic tool that is readily available, safe, comparable accuracy, relatively inexpensive and ability to improve pregnancy rate (especially when oil-based contrast media are used for the procedure) [4,5].

The pathologies detected on HSG may classified tubal, peritubal, uterine/intracavitory, and cervical pathologies [5-7]. The tubal abnormalities/pathologies detected on HSG include tubal occlusion/blockage, which may be due to spasm; infection; or congenital anomalies. Tubal occlusion manifests as non-opacification of the entire fallopian tube or opacification of the proximal fallopian tube and an abrupt cutoff of contrast media with non-opacification of the distal fallopian tubes. Therefore, tubal pathologies can involve the proximal, mid, or distal parts of the fallopian tube and the tubal occlusion can be unilateral (left or right) or bilateral. Peritubal adhesions prevent contrast media from spilling into the peritoneal cavity and distributing freely. Uterine pathologies can manifest as filling defects or irregular outlines of the uterine cavity. A well-defined filling defect suggests submucosal leiomyoma or endometrial polyp, while ill-defined filling defect suggests intrauterine adhesion or synechiae. The absolute contraindications for HSG are pregnancy, active pelvic infection, recent tubal or uterine surgery, and active vaginal or uterine bleeding. Although HSG is relatively safe procedure, it may be complicated by pelvic cramps/pain, pelvic infection, fever, nausea, vaso-vagal symptoms or even lymphogranuloma formation [5-7]. Despite the potential complications and disadvantages such as exposure to radiation and high false positive rate, HSG still remains one of the most commonly used imaging modality for evaluating female infertility in most countries such as Nigeria, United States and United Kingdom [8]. In addition to HSG, the other tools deployed in the evaluation of females with infertility include two- and three-dimensional pelvic ultrasonography, sonohysterosalpingography, hysteroscopy, laparoscopy, and magnetic resonance imaging (MRI) [6,9].

## Methods

This study was carried out at the fertility clinic (reproductive endocrinology and fertility regulation unit) of the Lagos University Teaching Hospital (LUTH). LUTH is the teaching hospital of the College of Medicine, University of Lagos. It acts mainly as a referral center for other government-owned and private hospitals in Lagos State. The hospital offers specialized treatment to infertile couple seeking care including in-vitro fertilization. This study was a retrospective review of medical records of women seeking treatment for infertility over a two-year period, between January 2018 and December 2019. All the medical records of infertile couples with HSG were retrieved and reviewed including the HSG report and film. The relevant parameters/variables of interest were collected using a structured questionnaire. The information sought included uterine and cervical outline (presence or absence of filling defects), and tubal patency or occlusion. Data were entered, cleaned, and analyzed using the IBM Statistical Package for Social Sciences (SPSS Statistics) Version 23. Armonk, NY: IBM Corp. The categorical variables were summarized and presented as frequency distribution tables, while continuous variables were presented as mean (standard deviation). A binary regression was conducted to investigate factors that predict the presence of tubal pathologies on hysterosalpingography (HSG). The dependent variable of interest on dichotomous outcome was the absence or presence of an abnormal tubal finding on HSG; while the possible predictors were age, duration of infertility, previous history of pelvic infections and surgeries such as myomectomy, ovarian cystectomy, caesarean section, and uterine evacuation. The dichotomous outcome was assigned “0” and “1” for absence and presence of abnormalities or pathologies on HSG, respectively. The predictor variables were assigned “0” and “1” for “No” and “Yes” responses to previous history of pelvic infection/surgeries, respectively. P value less than 0.05 was considered statistically significant.

## Results

A total of 296 medical records and report of HSG results were reviewed, 266 had complete data set and were included in the data analysis. The mean age (±SD) was 38.4 (±0.3) years with a range of 24 to 50 years. Majority (65.4%) of the participants were nulliparous and most (80.5%) of them have secondary infertility. The mean duration (±SD) of infertility was 5.4 (±3.4) years with a range of one to twenty-one years. Two hundred and fourteen women (80.5%) had secondary infertility while 52 (19.5%) had primary infertility. The mean (±SD) duration of infertility in our study population was 5.4 (±3.4) years. About one-fifth of the study participants have had previous myomectomy. Table 1 shows the baseline characteristics of study participants. Table 2 shows the radiological findings on hysterosalpingography. About one-third (34.6%) of participants had normal patent tubes, while 174 (65.4%) of participants had at least one tubal abnormality on HSG. Eighty-two participants (30.4%) had bilateral tubal occlusion. The proportion of participants with right tubal occlusion was 43.2%, while the proportion with left tubal occlusion was 41.7%. About one-tenth (11.7%) of participants had well-defined filling defects suggestive of uterine fibroids. Table 3 shows the logistic regression model of potential predictors of tubal pathologies on HSG. The model showed that the independent variables: duration of infertility; history of previous pelvic inflammatory disease; and history of previous caesarean section and ovarian cystectomy were not significant (p-value > 0.05). However, the independent variables age (OR 1.055; 95% CI: 1.006, 1.106, p-value 0.028), previous myomectomy (OR 4.6; 95% CI: 1.34, 28.35, p-value 0.02) and salpingectomy (OR 6.15; 95% CI: 1.81, 11.67, p-value 0.001) were found to be significant.

**Table 1:** baseline characteristics of study participants (N = 266)

Variables	Frequency n (%)	Mean (SD)
Age group (years)		
≤ 30	29 (10.9)	
31 - 35	60 (22.6)	
36 - 40	81 (30.4)	
41 - 45	54 (20.3)	
46 - 50	42 (15.8)	
Mean age (years)		38.4 (0.3)
Parity		
0	174 (65.4)	
1	71 (26.7)	
≥ 2	21 (7.9)	
Type of infertility		
Primary	52 (19.5)	
Secondary	214 (80.5)	
Mean duration of infertility (years)		5.4 (3.4)
Potential risk factors for tubal pathologies		
Caesarean section	22 (8.3)	
Myomectomy	59 (22.2)	
Cystectomy	15 (5.6)	
Salpingectomy	25 (9.4)	
Manual vacuum aspiration	37 (13.9)	
Pelvic inflammatory disease	41 (15.4)	

**Table 2:** radiological findings on hysterosalpingography (N = 266)

Findings on hysterosalpingography	Frequency n (%)
**Fallopian tubes**	
Left fallopian tube	
Blocked	111 (41.7)
Hydrosalpinx	27 (10.2)
Loculated spill	5 (1.9)
Patent	120 (45.1)
**Right fallopian tube**	
Blocked	115 (43.2)
Hydrosalpinx	24 (9.0)
Loculated spill	5 (1.9)
Patent	120 (45.1)
**Both fallopian tubes**	
Blocked	82 (30.8)
Patent	92 (34.6)
Peritoneal abnormalities	
Perifimbrial adhesions	33 (12.4)
**Uterus**	
Bicornuate	2 (0.8)
Hypoplastic	3 (1.1)
Irregular outline	4 (1.5)
Well-defined filling defects	31 (11.7)
Ill-defined filling defects	14 (5.3)
**Cervix**	
Irregular	5 (1.9)
Patulous	5 (1.9)
Stenosis	2 (0.8)

**Table 3:** logistic regression model of potential predictors of tubal pathologies on hysterosalpingography (N = 266)

Predictors	β	OR	95% CI for OR		P-value
			Lower	Upper	
Constant	-1.745	0.175	-	-	0.048
Age (years)	.053	1.055	1.006	1.106	0.028
Previous myomectomy	1.526	4.600	1.814	11.670	0.001
Previous salpingectomy	1.817	6.151	1.335	28.349	0.020
Previous PID	0.881	2.414	0.749	7.782	0.140
Previous ovarian cystectomy	0.905	2.472	0.607	10.077	0.207
Previous caesarean sections	-0.407	0.666	0.235	1.884	0.443
Duration of Infertility (years)	-0.014	0.986	0.904	1.075	0.747

OR = odds ratios, CI = confidence interval, β = logit coefficient, PID = pelvic inflammatory disease

## Discussion

Hysterosalpingography still serves as an invaluable tool in the investigation of women with infertility in modern obstetrics practice despite the advent of laparoscopy and dye test. It is invaluable especially in resource poor settings like ours where laparoscopy and dye test is not readily available or when available, the cost is prohibitive. There is therefore, a need for the review of HSG findings in women with infertility. Tubal abnormality was the commonest radiological finding on the HSG of female partners of infertile couples seeking treatment at LUTH. Out of the 266 participants, about two-thirds had at least one form tubal abnormality on HSG and about one-third had radiological evidence suggestive of bilateral tubal occlusion. Previously, there was a debate as to whether HSG is a reliable tool for detection of tubal patency or tubal blockage. The various reports on the test accuracy of HSG are mixed and inconsistent. However, available evidence suggests that up to 60% of cases diagnosed with tubal obstruction using HSG have been shown via laparoscopy to have patent fallopian tubes [10-13].

In addition, a systematic review by Maheux-Lacroix *et al*. found that 80% of patients with an HSG diagnosis of bilateral proximal obstruction were subsequently shown using sonohysterography to have at least one patent tube [9]. In this study, the proportion of participants with right fallopian tube occlusion was relatively higher when compared to left tubal occlusion (43.2% versus 41.7%). Available data show that occurrence of fallopian tube abnormality appears to be commoner on the right when compared to the left fallopian tube [14-16]. This finding has been attributed to previous appendicitis and/or appendectomy with its surgical complications. Ninety-two (34.6%) of participants had normal patent tubes with free spillage and distribution of the contrast medium, while 12.4% had loculated spill suggestive of perifimbrial adhesion. Hydrosalpinx was found to be commoner on the left (10.2%) compared to the right and this is similar to the finding by Onwuchekwa *et al*. [14]. However, this was different from the finding of Bukar *et al*. who reported right preponderance of hydrosalpinx in comparison to the right [17]. Perifimbrial/peritubal adhesions suggest pelvic adhesion either from previous surgeries, poorly treated pelvic inflammatory disease, or even endometriosis. Suspected pelvic adhesions accounted for 12.4% of the abnormalities detected on HSG in our study. Similar studies have also reported features suggestive of perifimbrial or peritubal adhesions [3,7,18].

Although some studies have reported primary infertility as the predominant type among women who had HSG for evaluation of infertility [7,19], secondary infertility was the most predominant type of infertility in this study. Similar studies in in Nigeria [4,14,15] and other parts of the world [19] have reported secondary infertility as the commonest type among female partners of couples with infertility undergoing HSG. This finding may be attributed to the prevalent cause of female infertility in the study population such as pelvic infection or pelvic inflammatory disease. Submucous fibroid was the commonest uterine abnormality detected in this study and about one-fifth of the HSG showed features suggestive of uterine fibroids. Uterine fibroid is the commonest benign uterine tumour in females, especially black women of reproductive age. Uterine synechiae was the next commonest uterine abnormality noted on HSG. This finding has been attributed to infectious morbidities and excessive/overzealous uterine curettage [17]. Only five cases (1.8%) of uterine congenital anomaly was reported in our study which is higher than the 0.4% and 0.6% reported by Onwuchekwa *et al*. [14] and Danfulani *et al*. [15] respectively. Bukar *et al*. [17] reported a higher incidence of congenital uterine anomaly of 3.6%. In this study, the cervical abnormalities detected on HSG include irregular cervical outline, patulous cervix, and cervical stenosis. These types of cervical abnormalities have also been reported by Udobi *et al*. [7] and Bukar *et al*. [17].

The major risk factors identified for the presence of tubal pathology in our study were previous myomectomy and previous salpingectomy. Our finding may be attributed to the prevalence of secondary infertility in the study population and the proportion who had previous pelvic and uterine surgeries. This finding is consistent with the report of Lawan *et al*. who also reported prior pelvic surgery as the commonest risk factor for tubo-peritoneal abnormality in their study [20]. In contrast, some studies reported previous pelvic inflammatory disease as the most common risk factor for tubal blockade/abnormalities [21,22]. Tubal abnormalities ranging from occlusion, hydrosalpinx and loculated spill accounted for the greatest abnormalities seen in our study. This is consistent with others studies where tubal abnormalities were reported on HSG [21-23].

**Limitation:** this is a retrospective data, in which few patients´ records were not complete, therefore were not included in the data analysis.

## Conclusion

The role of HSG as a screening tool in women with suspected tubal factor infertility in a low resource setting cannot be overemphasized as its important in excluding tubal occlusion or abnormalities, and may serve as a basis for further tubal patency assessment. Previous myomectomy and salpingectomy are identifiable risk factor for the presence of tubal pathologies.

### What is known about this topic

- It is a fact that tubal damage contributes to female factor infertility, especially in developing countries;
- hysterosalpingography on the other hand serves as a good screening tool for tubal patency assessment in evaluating female partners of infertile couple with suspected tubal factor infertility.

### What this study adds

- Tubal pathology was the commonest abnormality detected on HSG, even among nulliparous women.
- The women´s age and previous surgeries (myomectomy and salpingectomy) were identifiable risk factors for tubal pathologies on HSG.
- One-third of women had bilateral tubal occlusion on HSG and might require assisted conception technology for treatment of infertility.
